# Composition and biological activities of the aqueous extracts of three scleractinian corals from the Mexican Caribbean: *Pseudodiploria strigosa*, *Porites astreoides* and *Siderastrea siderea*

**DOI:** 10.1186/s40409-016-0087-2

**Published:** 2016-11-24

**Authors:** Alejandro García-Arredondo, Alejandra Rojas-Molina, César Ibarra-Alvarado, Fernando Lazcano-Pérez, Roberto Arreguín-Espinosa, Judith Sánchez-Rodríguez

**Affiliations:** 1 Departamento de Investigación Química y Farmacológica de Productos Naturales, Facultad de Química, Universidad Autónoma de Querétaro, Querétaro, 76010 Mexico; 2 Departamento de Ciencias de la Salud, Universidad Autónoma Metropolitana, Campus Iztapalapa, Mexico City, 09340 Mexico; 3 Instituto de Química, Universidad Nacional Autónoma de México, Mexico City, 04510 Mexico; 4 Unidad Académica de Sistemas Arrecifales, Instituto de Ciencias del Mar y Limnología, Universidad Nacional Autónoma de México, Puerto Morelos, Quintana Roo 77500 Mexico

**Keywords:** Scleractinia, *Pseudodiploria strigosa*, *Porites astreoides*, *Siderastrea siderea*, Toxicity, Hemolysis, Vasoconstriction, Nociceptive response

## Abstract

**Background:**

Scleractinian corals (stony corals) are the most abundant reef-forming cnidarians found in coral reefs throughout the world. Despite their abundance and ecological importance, information about the diversity of their toxins and their biological activities is very scarce. In this study, the chemical composition and the biological activities of the aqueous extracts of *Pseudodiploria strigosa*, *Porites astreoides* and *Siderastrea siderea*, three scleractinian corals from the Mexican Caribbean, have been assessed for the first time.

**Methods:**

Toxicity of the extracts was assessed in crickets; the presence of cytolysins was detected by the hemolysis assay; the vasoconstrictor activity was determined by the isolated rat aortic ring assay; the nociceptive activity was evaluated by the formalin test. The presence of phospholipases A_2_ (PLA_2_), serine proteases, and hyaluronidases was determined by enzymatic methods. Low-molecular-weight fractions were obtained by gel filtration chromatography and ultrafiltration.

**Results:**

Extracts from the three species were toxic to crickets, induced hemolysis in human and rat erythrocytes, produced vasoconstriction on isolated rat aortic rings, and presented phospholipase A_2_ and serine-protease activity. Despite the fact that these corals are not considered to be harmless to humans, the extracts generated significant nociceptive responses. The matrix-assisted laser desorption/ionization time-of-flight (MALDI-TOF) mass spectrometry analysis of the low-molecular-weight fractions revealed the presence of peptides within a mass range of 3000 to 6000 Da. These fractions were toxic to crickets and two of them induced a transitory vasoconstrictor effect on isolated rat aortic rings.

**Conclusion:**

This study suggests that scleractinian corals produce low-molecular-weight peptides that are lethal to crickets and induce vasoconstriction.

## Background

Cnidaria is a phylum containing over 11,000 species of aquatic organisms with radial symmetry [1]; the vast majority occur in marine systems. At the present time, a well-accepted taxonomic classification of this phylum defines two groups. One group, the subphylum Meduzoa, includes the classes Scyphozoa (true jellyfish), Cubozoa (box jellyfish), Staurozoa (stalked jellyfish), Hydrozoa (hydroids), and Polypodiozoa (an endocellular parasite). The other group, the class Anthozoa, includes the subclasses Octocorallia (soft corals and sea pens) and Hexacorallia (sea anemones, zoanthids, and stony corals) [1].

All cnidarians are equipped with specialized stinging cells called nematocytes. Each nematocyte contains a specialized organelle referred to as a nematocyst, which is composed of a capsule filled with venom and an inverted tubule. Following an appropriate stimulation, the tubule is everted and injects the venom into the prey, such that nematocysts are predominantly used for prey capture and defense [2]. It is well-documented that the venomous content of nematocysts comprises mainly proteins and peptides such as enzymes, pore-forming components, and neurotoxins that specifically modulate several types of ion channels [3, 4]. Non-nematocystic toxins have also been found in cnidarian tissues [5, 6]; most of them are secondary metabolites that are usually released to their surroundings as antipredatory, antimicrobial, allelopathic, and antifouling agents [7].

Cnidarians are considered to be the largest phylum of generally toxic animals [3] and they are undoubtedly a very important source of novel bioactive natural products useful in the generation of new drug lead compounds or pharmacological tools for the study of cell physiology. In this regard, evaluation of the bioactivity of cnidarian extracts provides an overview of the properties of their compounds and their potential pharmacological applications. In addition, these evaluations are important, as they allow one to obtain information about the toxicity and the defensive mechanisms of cnidarians.

Despite the fact that most toxicological studies of cnidarians have focused on the class Anthozoa, the order Scleractinia remains relatively unexplored. Scleractinian corals, also called stony corals, are the most abundant reef-forming cnidarians found in coral reefs throughout the world [8]. In an earlier study, variable toxicity was found in several scleractinian corals from the Great Barrier Reef [9]. In the present study, the chemical composition and the biological activities of the aqueous extracts of three scleractinian corals collected in the Mexican Caribbean – *Pseudodiploria strigosa*, *Porites astreoides*, and *Siderastrea siderea* – were assessed for the first time. The biological activities evaluated were toxicity to crickets, hemolysis, vasoconstriction, and nociceptive activity. Furthermore, enzymatic activities such as phospholipase A_2_ (PLA_2_), serine protease, and hyaluronidase activity were investigated. Low-molecular-weight fractions were obtained from each extract; some biological activities of these fractions were assessed and also analyzed by matrix-assisted laser-desorption/ionization time-of-flight (MALDI-TOF) mass spectrometry.

## Methods

### Laboratory animals

The animals were permitted free access to standard mouse food pellets and water *ad libitum*. All experiments were performed in accordance with the Official Standard NOM-062-ZOO-1999 for the production, care, and use of laboratory animals. The care and use of the animals was approved by the Bioethics Committee of the School of Medicine, UAQ.

### Materials

Sodium citrate, citric acid, L-phenylephrine, formalin, N-benzoyl-arginine-p-nitroanilide, trypsin from porcine pancreases, hyaluronic acid sodium salt from *Streptococcus equi*, hexadecyltrimethylammonium, hyaluronidase from bovine testes type IV-S, and sinapinic acid (>99.0% purity) were obtained from Sigma (USA). Refined chemicals used for buffer preparations, deionized water, and HPLC grade water were purchased from J.T. Baker (USA). All reagents used in the determination of protein concentration and in electrophoretic analysis were obtained from Bio-Rad (USA).

### Specimen collection

Fragments of corals were collected by scuba diving at depths of 4 to 10 m from coral reefs along the coast at Puerto Morelos, Quintana Roo state, Mexico, in October 2013. The fragments were kept wet with sea water for their transportation to the laboratory where they were then frozen and stored at −70 °C. Specimen collection was conducted according to the guidelines of the National Commission of Aquaculture and Fishing, of the Secretariat of Agriculture, Livestock, Rural Development, Fishing, and Feeding of the Mexican Federal Government (permit number PPF/DGOPA-193/13).

### Aqueous extract preparation

Nematocyst discharge of each species was induced by stirring the calcareous fragments in HPLC grade water at 4 °C for 24 h. The extracts obtained were centrifuged at 3000 rpm (2060 × g) for 15 min at 4 °C and the supernatants were separated; this procedure was repeated twice. The supernatants were freeze-dried and dissolved in HPLC grade water at a concentration of 150 mg/mL and centrifuged at 3000 rpm for 15 min at 4 °C. The supernatants obtained were stored at −70 °C and used to perform the bioassays and other analyses. The protein content of the aqueous extracts was determined by the Bradford assay [10], using a standard curve prepared with lyophilized bovine serum albumin.

### Bioassays

#### Toxicity assay

In order to compare the toxicities of the aqueous extracts, they were assessed in crickets (*Acheta domestica*) of undetermined sex that weighed 190–210 mg by a method previously described [11]. Briefly, lyophilized extracts were dissolved in insect saline solution (200 mM NaCl, 3.1 mM KCl, 5.4 mM CaCl_2_, 4 mM MgCl_2_, 2 mM NaHCO_3_, 0.1 mM Na_2_HPO_4_; pH 7.2) and administrated by thoracic injection into crickets (five crickets per dose) at several doses (1, 3.2, 10, 31.6, 100, and 316 μg protein/mL). The injection volume for all crickets, including the controls that received insect saline solution, was 10 μL. Injections were performed using a 0.3 mL insulin syringe (BD Ultra-Fine™, Terumo Medical Corporation, Elkton, MD, USA). After the injection, the crickets were placed in small plastic containers with food and water *ad libitum*. Mortality was scored at 24 and 48 h post-injection. The median lethal dose (LD_50_) values were interpolated by fitting log dose-response curves (*n* = 3/curve) using non-linear regression analysis.

#### Hemolysis assay

In order to determine the presence of cytolysins in the extracts, the hemolytic activity in human and rat erythrocytes was monitored according to a previously reported method [12]. Briefly, an aliquot of blood was washed three times with Alsever’s solution (120 mM D-glucose, 30 mM sodium citrate, 7 mM NaCl, and 2 mM citric acid; pH 7.4). Washing was done by low-speed centrifugation at 2500 rpm (1430 × g) for 4 min at 4 °C. Different concentrations of the aqueous extracts (0.003, 0.010, 0.032, 0.100, 0.316, 1, 3.16, 10, 31.6, and 100 μg of protein/mL) were combined with the washed erythrocytes (1%) and Alsever’s solution to comprise a total volume of 1 mL. Next, the samples were incubated at 37 °C for 30 min. Then the samples were centrifuged at 2500 rpm (1430 × g) for 4 min.

The A_450_ of the supernatant containing the hemoglobin released from lysed erythrocytes was measured in a spectrophotometer. Each experiment was normalized with respect to complete hemolysis, which was induced by diluting the erythrocyte sample in deionized water instead of Alsever’s solution. One hemolytic unit (HU_50_) was defined as the amount of protein sample required to induce 50% hemolysis in a 1% erythrocyte solution at 37 °C for 30 min. The HU_50_ values were interpolated by fitting log concentration-response curves (*n* = 3/curve) using non-linear regression analysis.

#### Isolated rat aortic ring assay

The assay was carried out in order to determine whether or not the extracts contain components that produce effects on the cardiovascular system [13]. Briefly, male rats (250–275 g) were sacrificed by decapitation and the descending thoracic aorta was removed and placed in ice-cold, oxygenated Krebs-Henseleit solution (126.8 mM NaCl, 5.9 mM KCl, 2.5 mM CaCl_2_, 1.2 mM MgSO_4_, 1.2 mM KH_2_PO_4_, 30 mM NaHCO_3_, and 5 mM D-glucose; pH 7.4). The aorta was immediately flushed with Krebs-Henseleit solution in order to prevent the formation of intravascular clots; it was dissected free of connective tissue and cut into rings at 4 to 5 mm intervals. The aortic rings were mounted between stainless steel hooks and suspended in 5 mL water-jacketed organ baths containing oxygenated (95% O_2_ and 5% CO_2_) Krebs-Henseleit solution at 37 °C. The tissues were allowed to equilibrate for 60 min under a resting tension of 1.5 g. During this period, the bathing medium was renewed every 15 min.

After a final adjustment of the passive resting tension to 1.5 g, aortic segments were contracted with 100 mM KCl. Once a stable contractile tone was reached, the bathing medium was renewed to restore a resting tension of 1.5 g. The tissues were then contracted with 1 μM L-phenylephrine, the force of contraction was recorded, and this contraction was set as 100%. The bathing medium was changed again to restore the original resting tension, and then the aqueous extracts were added to the organ bath. The isometric tension was measured by a Grass FT03 force-displacement transducer connected to a Grass 7D polygraph. The responses were expressed as a percentage of the initial contraction achieved with phenylephrine. The half maximal effective concentration (EC_50_) and the maximum effect (E_max_) values were interpolated by fitting log concentration-response curves (*n* = 3/curve) using non-linear regression analysis.

#### Formalin test

Acute neurogenic and chronic inflammatory nociception of the extracts were measured using the rat paw formalin test [14]. For this test, extracts (40 μg protein/paw, dissolved in 50 μL of sterile saline solution) were injected subcutaneously into the right dorsal hind paw of male Wistar rats weighing 100–120 g (*n* = 3/group). The positive control group received 50 μL of 2.5% formalin, while the negative group received 50 μL of saline solution. During the test, each rat was placed in a transparent glass container and nociceptive behavior was quantified by counting the time of licking, flinching, and lifting of the injected hind paw using stop-clocks. Measurements were made in two phases: in the first phase (neurogenic), the mice were evaluated from 0 to 10 min post-injection, and in the second phase (inflammatory) from 10 to 50 min post-injection. The animals were euthanized immediately at the end of the test. All experiments were video-recorded for backup purposes.

#### PLA_2_ assay

The PLA_2_ activity of the aqueous extracts was determined using a secretory PLA_2_ colorimetric assay kit (Cayman Chemical, USA). This assay uses the 1,2-dithio analog of diheptanoyl phosphatidylcholine as substrate. Free thiols generated by PLA_2_ upon hydrolysis of the thioester bond at the sn-2 position were detected using DTNB [5,5’-dithio-bis-(2-nitrobenzoic acid)]. Color changes were monitored by a Benchmark Plus microplate spectrophotometer at 414 nm, sampling every minute for 10 min. Ten microliters (10 μg) of bee venom PLA_2_ control was used as the reference for PLA_2_ activity. PLA_2_ activity was expressed in μmol of hydrolyzed phosphatidylcholine per minute per mg of protein (*n* = 3).

#### Serine protease assay

Serine protease activity of the extracts was determined by a method previously described [15]. This assay uses the N-benzoyl-arginine-p-nitroanilide as substrate (0.01 M, 10 μl); the rate of hydrolysis was assayed in 0.1 M Tris-HCl, pH 8. The yellow p-nitroanilide generated was monitored by a microplate spectrophotometer at 405 nm, sampling every two minutes for 60 min. Trypsin from porcine pancreas was used as the reference for serine protease activity. Serine protease activity was expressed in units (U)/mg of protein (*n* = 3); one unit of activity is equivalent to an absorbance increase of 0.01 per minute.

#### Hyaluronidase assay

Hyaluronidase activity was determined according to a turbidimetric method [16]. In short, different concentrations of venoms (1, 2.5, 5, 7.5, 10, 15, 20, and 25 μg/mL) diluted in 150 μL buffer (0.2 M sodium acetate, pH 6.0, containing 0.15 M NaCl), were incubated with 100 μL of substrate (1 mg of hyaluronic acid sodium salt from *Streptococcus equi* in 1 mL of acetate buffer) at 37 °C for 15 min.

After the incubation period, 1 mL of hexadecyltrimethylammonium (2.5%) in 2% NaOH was added to the samples. The resulting turbidity was read at 400 nm in a microplate spectrophotometer after 30 min of incubation at room temperature. Hyaluronidase from type IV-S bovine testes was used as the reference for hyaluronidase activity. Enzymatic activity was expressed in turbidity reducing units (TRU)/mg of protein (*n* = 3); one unit of activity corresponded to the amount of enzyme that produced a 50% reduction in turbidity caused by 200 μg of substrate under the conditions described above.

#### Data analysis and statistics

Data and statistical analyses were performed in Prism 5.0 (GraphPad Software, Inc., USA), and all results were expressed as means ± standard error of the mean (S.E.M.), of three replicates. The LD_50_, HU_50_, and EC_50_ values were interpolated by fitting log dose-response curves using non-linear regression analysis. Multiple comparisons were made by a one-way analysis of variance (ANOVA), followed by a *post hoc* Tukey’s test. In the case of the formalin test, the ANOVA was followed by a Dunnett test. In all cases, statistical significance is indicated by *p* < 0.05.

### SDS-polyacrylamide gel electrophoresis (SDS-PAGE)

Electrophoretic analyses of the extracts were performed as previously described under native and reducing conditions [17]. Under native conditions, samples were diluted 1:1 in a sample buffer (Bio-Rad, cat #161-0737). Samples under reducing conditions were diluted 1:1 in a sample buffer containing β-mercaptoethanol and heated at 95 °C for 5 min. Then, 12% polyacrylamide gels, loaded with 15 μg of protein, were electrophoresed at 120 V for 2 h at 4 °C, using Tris-Glycine as buffer (25 mM Tris, 192 mM glycine, pH 8.3, Bio-Rad, cat #161-0734). Protein bands were visualized using Coomassie brilliant blue R-250 staining solution (Bio-Rad, cat #161-0437), and molecular masses were determined by comparison with a broad-range polypeptide standard (Bio-Rad, cat #161-0318).

### Fractionation of extracts

For the fractionation, the extracts were passed through a fast performance liquid chromatography column (Sephadex G-50, 1.0 × 48 cm) coupled to an ÄKTA purifier system (UPC 100, GE Healthcare). The samples (500 μg of protein/500 μL) were eluted with 1.6 mM acetic acid at a flow rate of 1 mL/min. The absorbance was read at 280 nm and fractions (10 mL) were collected and pooled according to the peaks observed in the chromatogram. Then, the peaks retained in the column were filtrated with Amicon® Ultra centrifugation filters with 10 K cutoff (Merck Millipore, USA). Spin filtration was executed as recommended by the manufacturer manual supplied with the product. The chosen centrifugation time was 20 min at 14,000 × g.

### Mass spectrometry analysis

The fractions of low molecular mass were analyzed using matrix-assisted laser desorption/ionization time-of-flight (MALDI-TOF) mass spectrometry. Five microliters of a saturated solution of sinapinic acid was added to 10 μg of lyophilized fraction. Then, 1 μL of this solution was deposited onto the MALDI plate and allowed to dry at room temperature. The spectrum was recorded in linear positive mode on a mass spectrometer (Microflex Bruker Daltonics, Germany) equipped with nitrogen laser λ = 377 nm and 20 kV acceleration voltage.

### Microscopic examination of nematocysts

Samples of soft tissues of the corals were obtained using a 0.3 mL insulin syringe (BD Ultra-Fine™, Terumo Medical Corporation, USA) and were observed directly through light microscopy in order to identify the most abundant types of nematocysts in these corals.

## Results

### Aqueous extracts

The aqueous extracts prepared from the three corals showed similar protein concentrations. *P. strigosa* extract had a protein concentration of 10.8 mg/g of lyophilized powder; the extract obtained from *P. astreoides* showed a protein concentration of 12.9 mg/g; and 1 g of lyophilized extract of *S. siderea* contained 10.8 mg of protein.

### Bioassays

**Fig. 1:**
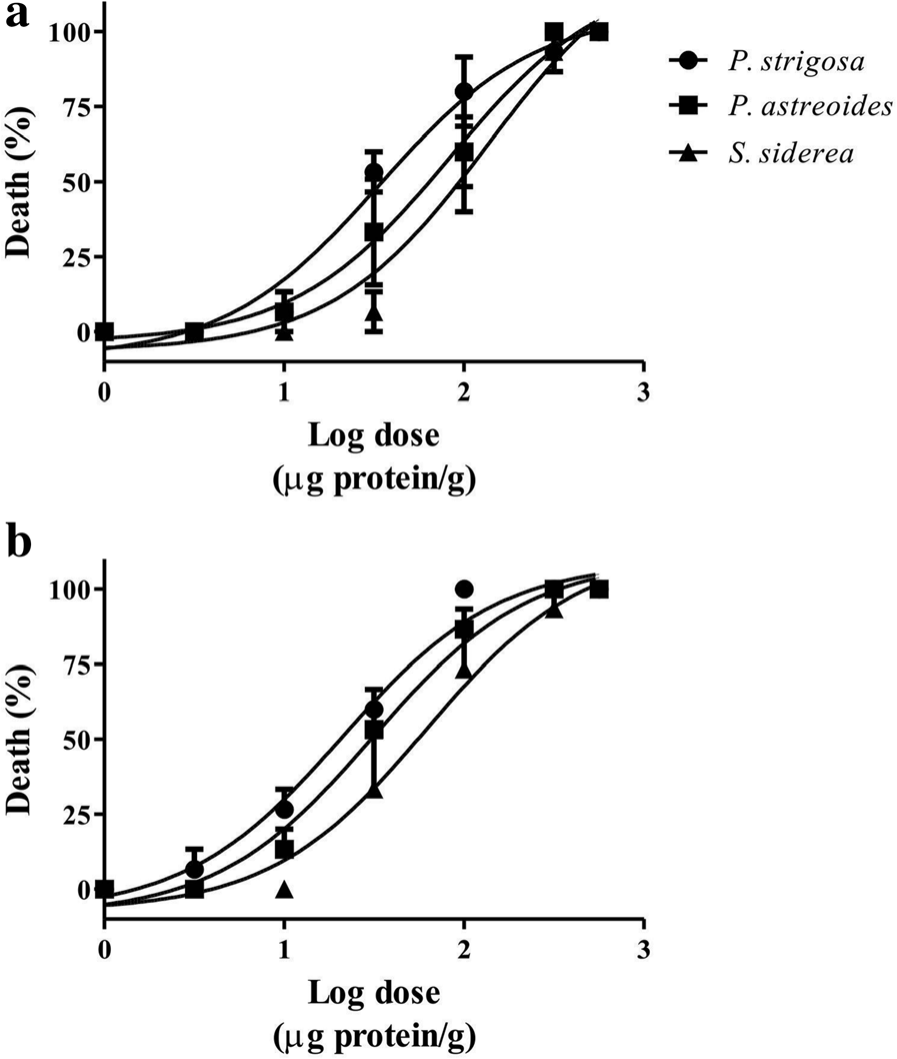
Toxicity of the extracts to crickets (*A. domestica*). Log-dose response curves at (**a**) 24 h and (**b**) 48 h post-injection

The insecticidal activity results showed that all extracts were lethal to crickets with similar potency and their lethality did not increase significantly with time. It is important to mention that the potency of the *S. siderea* extract appeared to increase with time; however, the ANOVA followed by Tukey’s test did not show significant differences. Figure 1 shows the dose-response curves while Table 1 displays the LD_50_ values. The extracts did not induce immediate paralysis, but at higher concentrations motility was gradually reduced.

**Table 1:** Toxicity of the aqueous extracts to crickets expressed as LD_50_ (μg protein/g)

	24 h post-injection	48 h post-injection
*P. strigosa*	33.99 ± 0.64	21.13 ± 2.54
*P. astreoides*	37.91 ± 12.03	33.25 ± 8.29
*S. siderea*	156.7 ± 49.07	37.35 ± 2.18

**Fig. 2:**
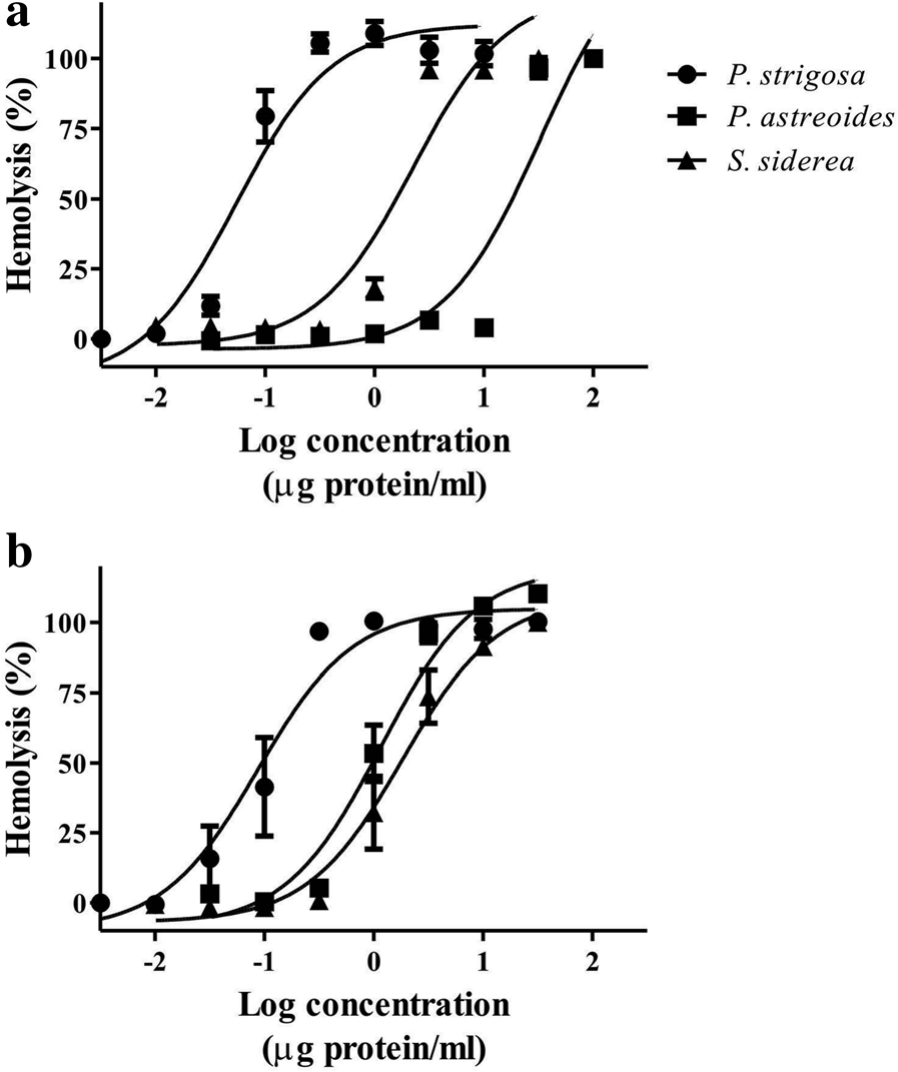
Log-concentration response curves showing the hemolytic activity of the extracts in (**a**) rat and (**b**) human erythrocytes

All the extracts induced concentration-dependent hemolytic activity in rat and human erythrocytes (Fig. 2). Table 2 shows HU_50_ values calculated for the extracts in both types of erythrocytes. The results obtained from the evaluation of rat erythrocytes showed that *P. strigosa* extract was significantly more potent than the others. When the extracts were evaluated in human erythrocytes, it was observed that they induced similar hemolytic activity without significant differences. The *P. astreoides* extract exhibited a low potency in rat erythrocytes, but significantly greater potency in human erythrocytes.

**Table 2:** Hemolytic activity of the aqueous extracts in rat and human erythrocytes expressed as HU_50_ (μg protein/mL)

	Rat erythrocytes	Human erythrocytes
*P. strigosa*	0.059 ± 0.015	0.107 ± 0.042
*P. astreoides*	32.43 ± 0.955*	3.373 ± 1.445^†^
*S. siderea*	2.476 ± 0.337	3.602 ± 0.987

^†^Significant difference (*p* < 0.001) compared to the activity in both types of erythrocytes

*Significant difference (*p* < 0.05) compared among the extracts

**Fig. 3:**
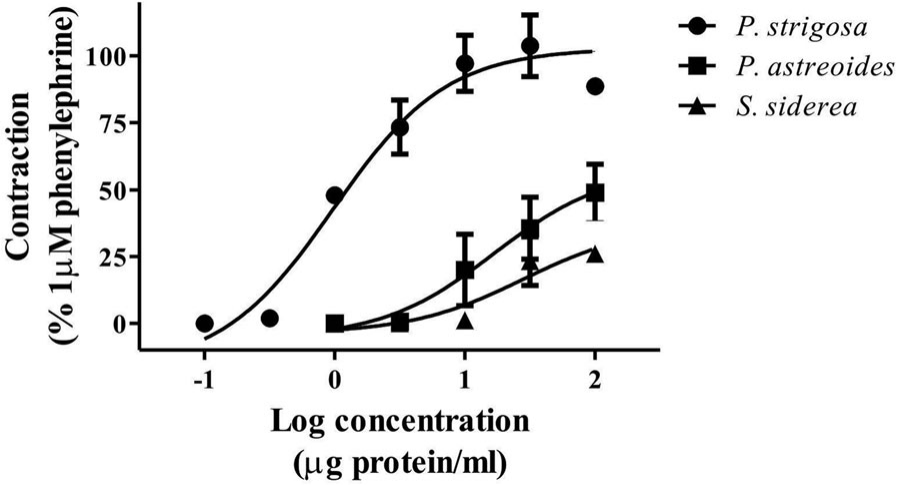
Log-concentration response curves showing the vasoconstrictor activity of the extracts

Assessment of the effect of the extracts on the vascular tone of isolated rat aortic rings showed that they induced a vasoconstrictor effect (Fig. 3). Table 3 shows the EC_50_ and E_max_ values calculated for the extracts. It is important to mention that the activity of the extracts was irregular; at concentrations lower than 10 μg of protein/mL the vasoconstriction was sustained, but at a higher concentration (approximately 100 μg of protein/mL) the vasoconstriction became transitory. At a concentration of 316 μg of protein/mL, the vasoconstriction induced by the extracts decreased considerably. These irregularities could be due to the presence of mixtures of vasorelaxant and vasoconstrictor components in the extracts. Comparisons between the activities induced by the extracts showed that *P. strigosa* extract was more potent and effective.

**Table 3:** Vasoconstrictor activity of the aqueous extracts in isolated rat aortic rings

	EC_50_ (μg protein/mL)	E_max_ (% Response)
*P. strigosa*	0.983 ± 0.17*	102.8 ± 5.63*
*P. astreoides*	16.97 ± 5.91	58.37 ± 15.94
*S. siderea*	28.22 ± 9.17	36.91 ± 11.00

*Significant difference (*p* < 0.05) compared among the extracts

**Fig. 4:**
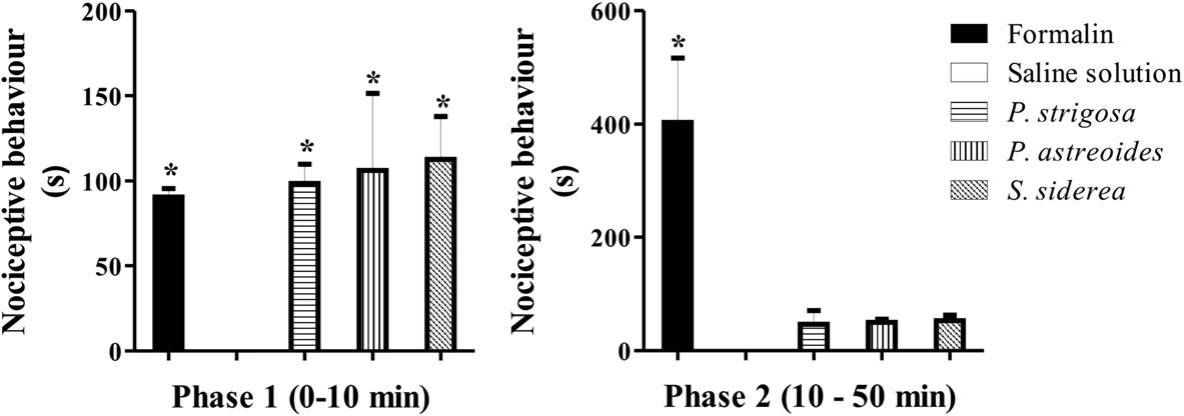
Formalin test for assessment of the nociceptive activity of the extracts in rats at a dose of 40 μg protein/paw. Nociceptive behavior in phase 1 (0–10 min post-injection) and phase 2 (10–50 min post-injection) was scored as the amount of time spent licking, flinching, and lifting. *Significant difference (*p* < 0.05) when compared to the negative group injected with saline solution

Evaluation of nociceptive activity by the formalin test showed that unilateral intraplantar injection of the extracts (40 μg of protein) generated significant nociceptive behavior only during the first phase (Fig. 4). As expected, formalin generated significant biphasic nociceptive behavior.

The results of the enzymatic activity showed that all extracts exhibited PLA_2_ and serine protease activities, but they did not display hyaluronidase activity. These results are summarized in Table 4.

**Table 4:** Enzymatic activity of the aqueous extracts

	PLA_2_ activity (μmol/min/mg)	Serine protease activity (U/mg)	Hyaluronidase activity (TRU/mg)
*P. strigosa*	0.03 ± 0.001	10.27 ± 1.99	–
*P. astreoides*	0.048 ± 0.002	11.81 ± 2.69	–
*S. siderea*	0.043 ± 0.001	3.95 ± 0.385	–
Control	21.268 ± 1.965	21.08 ± 1.857	149.51 ± 1.405

### SDS-PAGE analysis

**Fig. 5:**
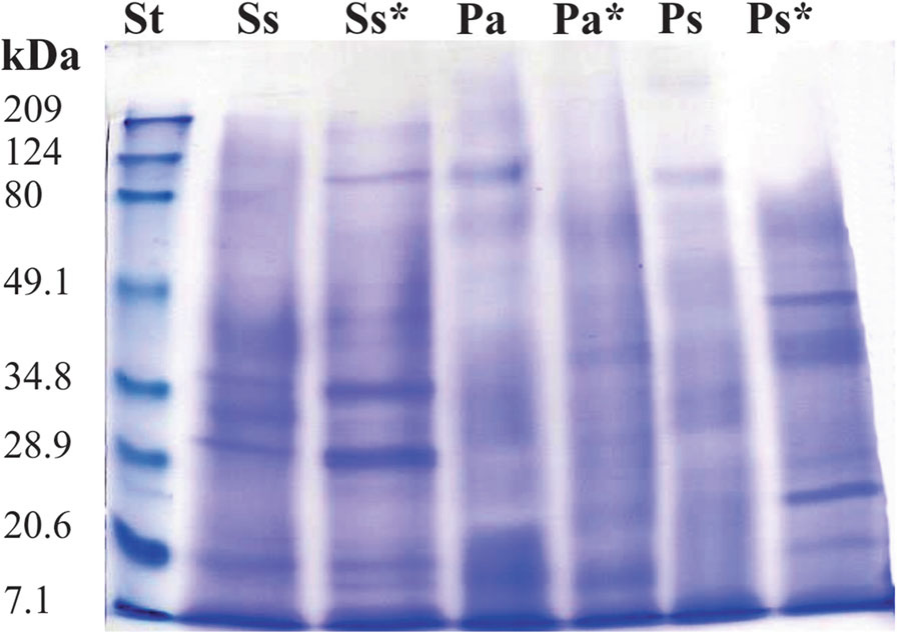
SDS-PAGE gel (12% acrylamide) showing an overview of the protein profiles of *S. siderea* (Ss), *P. astreoides* (Pa), and *P. strigosa* (Ps) extracts under native conditions. Ss*, Pa*, and Ps* correspond to the extracts under reducing conditions. The protein profiles were compared with a broad-range polypeptide standard (St). Protein bands were visualized with Coomassie brilliant blue staining solution

Analysis by electrophoresis afforded a preliminary overview of the protein components of the extracts, showing that the extracts contained such components in a broad range (Fig. 5). The profiles of each extract showed particular differences and changes when treated under reducing conditions.

### Composition and biological activities of low-molecular-weight fractions

**Fig. 6:**
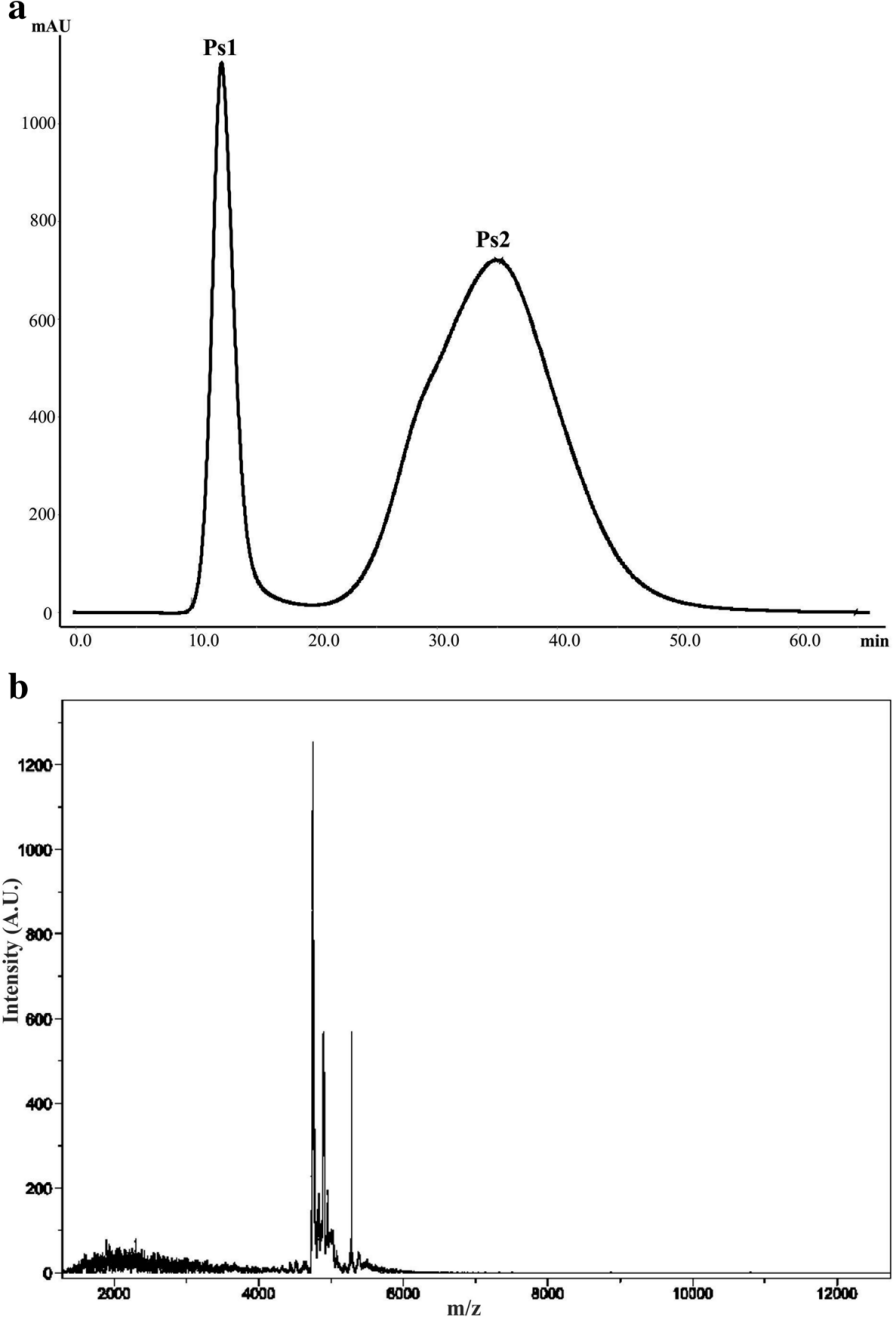
**a** *P. strigosa* extract elution profile obtained by FPLC on a Sephadex G-50 column at 280 nm. The column was equilibrated and eluted with 1.6 mM acetic acid at 1 mL/min. **b** MALDI-TOF of the second fraction (Ps2) after filtration with ultra-centrifugation filters with 10 K cut-off

**Fig. 7:**
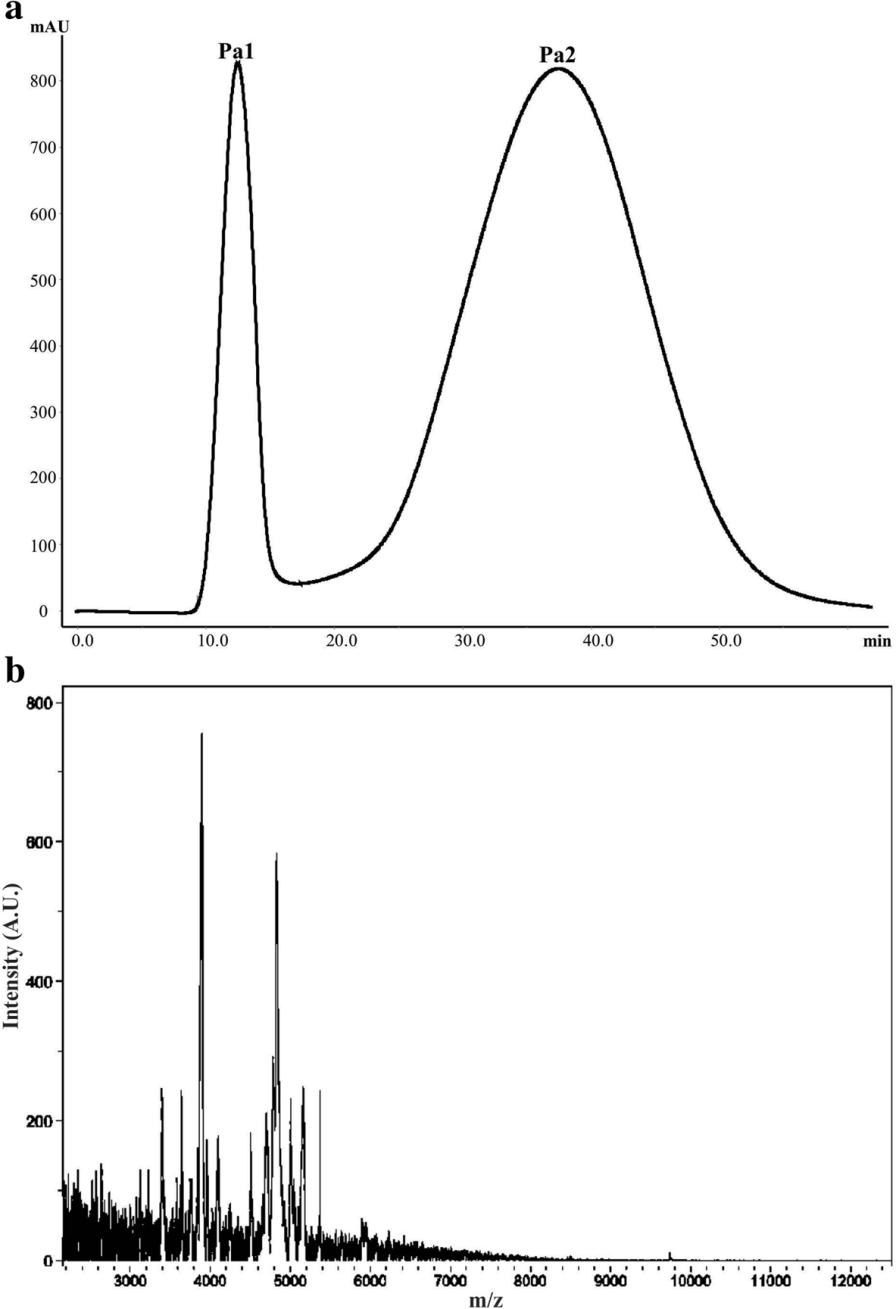
**a** *P. astreoides* extract elution profile obtained by FPLC on a Sephadex G-50 column at 280 nm. The column was equilibrated and eluted with 1.6 mM acetic acid at 1 mL/min. **b** MALDI-TOF of the second fraction (Pa2) after filtration with ultra-centrifugation filters with 10 K cut-off

**Fig. 8:**
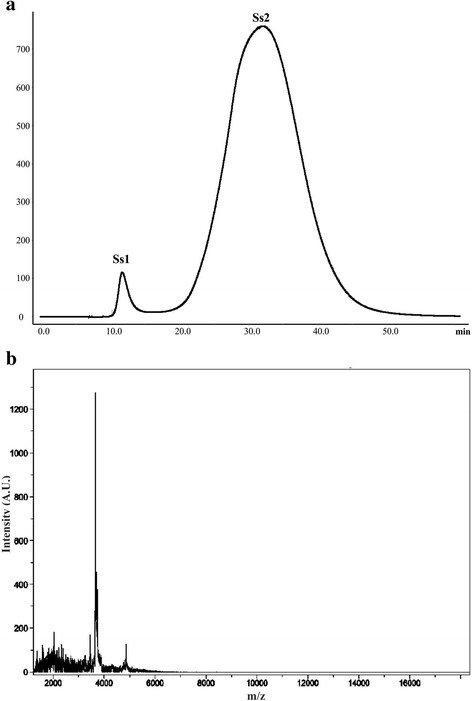
**a** *S. siderea* extract elution profile obtained by FPLC on a Sephadex G-50 column at 280 nm. The column was equilibrated and eluted with 1.6 mM acetic acid at 1 mL/min. **b** MALDI-TOF of the second fraction (Ss2) after filtration with ultra-centrifugation filters with 10 K cut-off

**Fig. 9:**
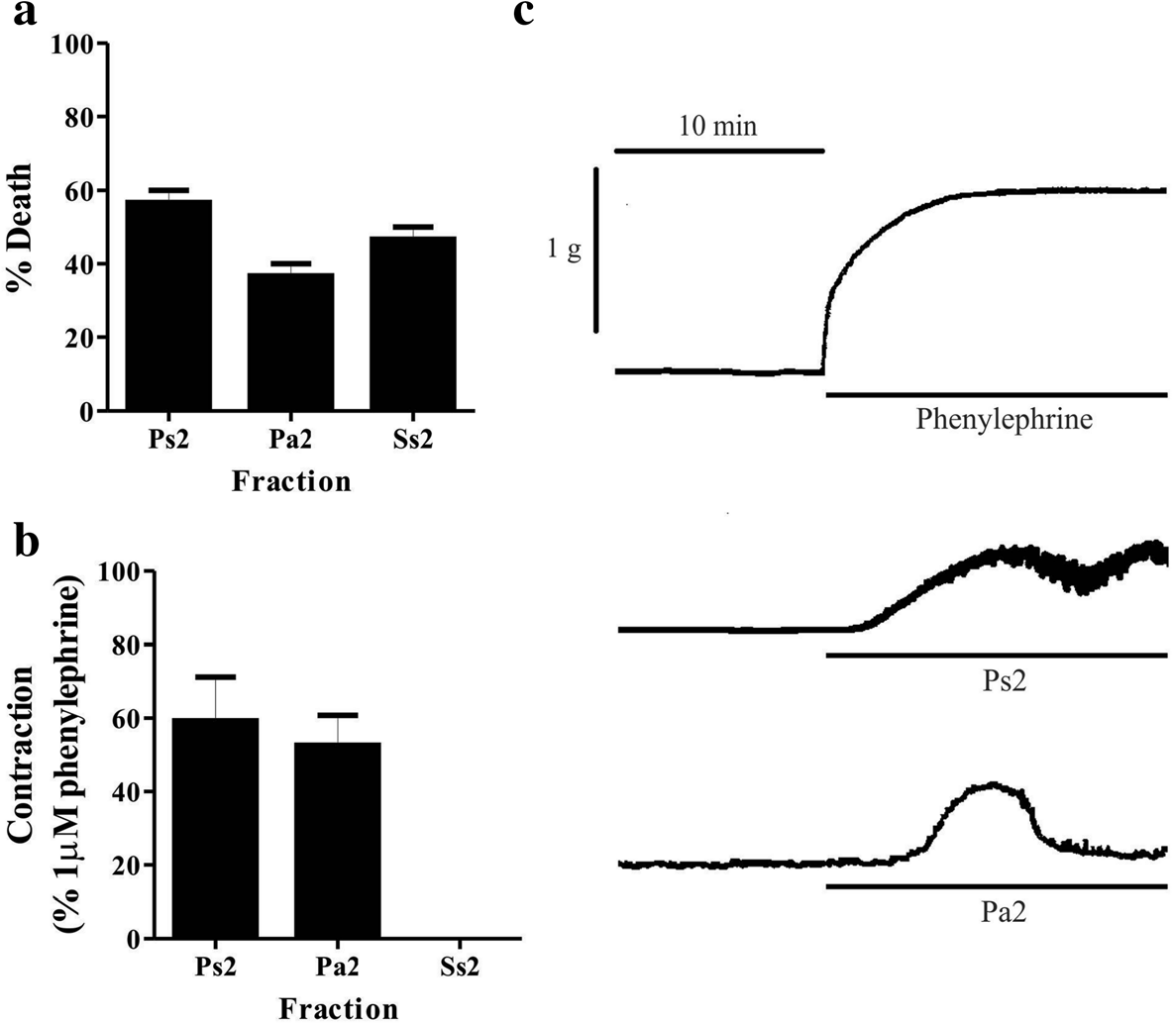
Biological activities of the low-molecular-weight fractions obtained from the aqueous extracts. **a** Toxicity to crickets (*A. domestica*), 24 h post-injection, at a concentration of 50 μg of lyophilized powder/g. **b** Vasoconstrictor activity on isolated rat aortic rings at a concentration of 1000 μg of lyophilized powder/mL. **c** Representative recordings showing the vasoconstrictor activity of the fractions (1000 μg/mL) compared with the activity induced by phenylephrine (1 μM)

Two fractions were separated from each extract using FPLC. The peaks retained in the column (Ps2, Pa2, and Ss2) were passed through filters with 10 K cutoff in order to obtain the low-molecular-weight components. The MALDI-TOF mass spectrometry showed that these fractions contain low-molecular-weight peptides, within a mass range of 3000 to 6000 Da (Figs. 6, 7, and 8). These low-molecular-weight fractions were lethal to crickets, 24 h post-injection, at a dose of 50 μg of lyophilized filtrated fraction/g (Fig. 9a). Evaluation of the vasoconstrictor activity of these low-molecular-weight fractions showed that Ps2 and Pa2 induced vasoconstriction at a concentration of 1000 μg of lyophilized filtrated fraction/mL, whereas Ss2 did not display activity (Fig. 9b-c).

### Observation of nematocysts

**Fig. 10:**
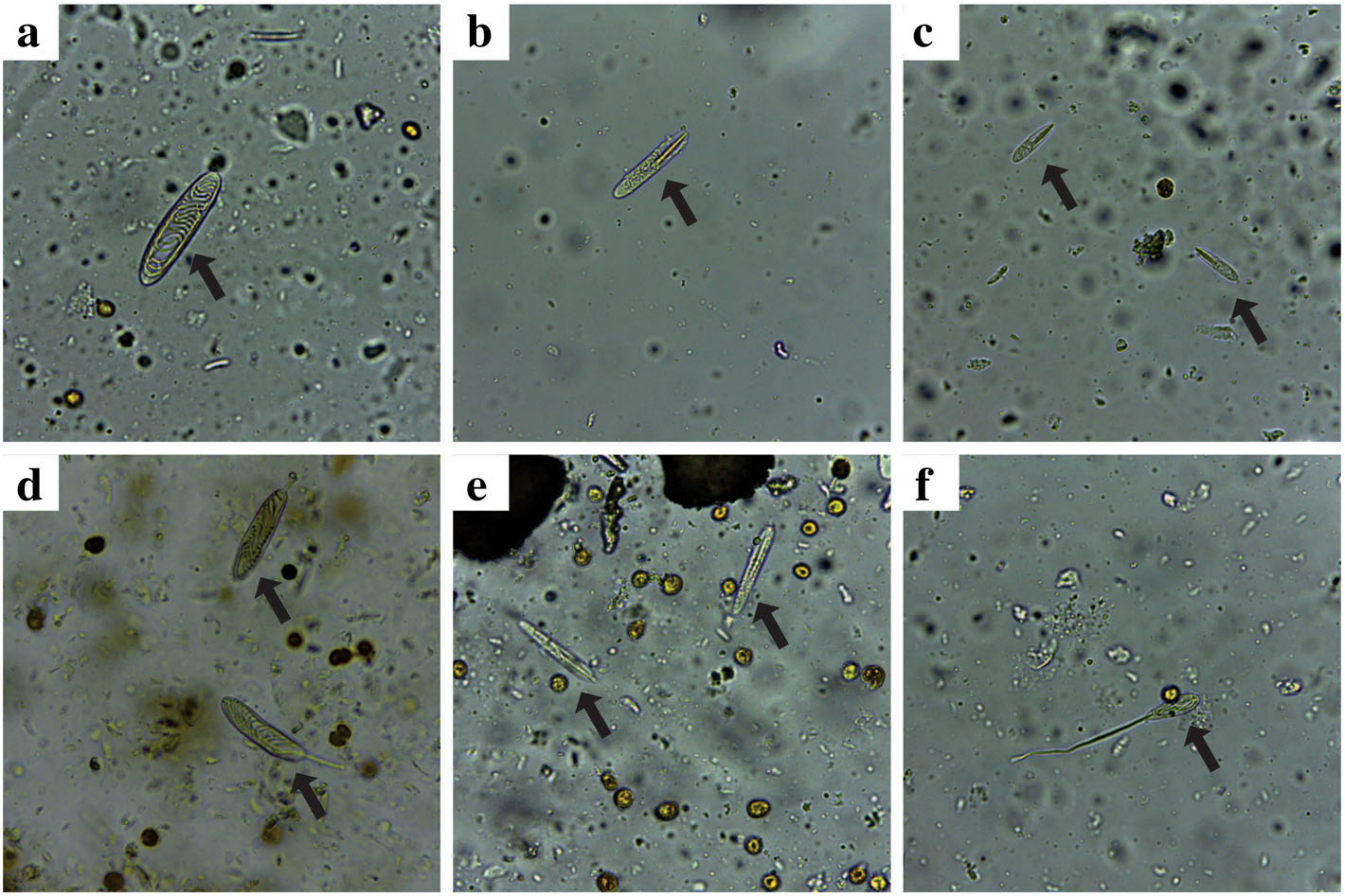
Light micrographs showing nematocyst found in soft tissues of the corals. **a** Holotrich of *P. strigosa*, (**b**) large p-rhabdoid of *P. strigosa*, (**c**) small p-rhabdoid of *P. strigosa*, (**d**) holotrich of *P. astreoides*, (**e**) p-rhabdoid of *S. siderea*, and (**f**) holotrich of *S. sidereal*. Magnification 400×

The microscopic examinations of soft tissues of the corals revealed the presence of two abundant types of nematocysts in *P. strigosa*, holotrich (Fig. 10a) and p-rhabdoid in two size classes (Fig. 10b-c). The most abundant type of nematocyst observed in *P. astreoides* tissues was holotrich (Fig. 10d), while p-rhabdoid (Fig. 10e) and some holotrich (Fig. 10f) were observed in *S. siderea* tissues.

## Discussion

In general, cnidarians are considered an important source of bioactive compounds, since this phylum has provided a great number of natural products with diverse structural properties. Many of these compounds could be used for the development of new drugs [3, 4, 18–21] or new pharmacological tools for the study of the basic molecular mechanisms of protein insertion into membranes and protein-lipid interactions [22], and new bioinsecticides [23, 24]. Many of the toxicological studies of cnidarians have focused mainly on electrophysiological analysis and cytolytic properties of anemone toxins (order Actiniaria, class Anthozoa). Other cnidarians have been extensively studied because of their toxicity to humans (classes Cubozoa, Scyphozoa, and Hydrozoa), so it is important to understand their mechanisms of envenomation in order to establish suitable treatments. However, despite their abundance in coral reefs, scleractinian corals have been poorly studied, which is probably due to the difficulty of separating soft tissues from the calcareous skeleton [9] and the fact that they are harmless to humans.

In this study, the bioactive properties of the aqueous extracts of three scleractinian corals collected in the Mexican Caribbean have been assessed for the first time. Considering that cnidarian venoms are composed mainly of peptides and proteins, the parameters of the biological activities of the extracts are expressed a quantity of protein [4]. However, it is possible that some secondary metabolites and other non-peptide components could contribute to the biological activities induced by the extracts. For this reason, the protein yield per gram of lyophilized powder is reported for each extract.

The extracts’ toxicity to crickets was determined in order to propose a simple bioassay that would allow an investigator to obtain comparative toxicological data. This assay is widely used to evaluate the toxicological activity of spider and scorpion venom because these animals feed on insects [11]. The natural preys of cnidarians are usually fish and crustaceans; in particular, scleractinian corals feed on small crustaceans and zooplankton [25]. However, the presence of insect-selective toxins in cnidarian venoms, mainly peptides affecting Na^+^ and K^+^ channels, has been reported and explained from an evolutionary point of view, in which it was proposed that insects are derived from crustacean ancestors [23].

In other studies, we have found that the aqueous extract from *Millepora complanata*, a reef-forming cnidarian of the class Hydrozoa, was lethal to crickets, with LD_50_ values of 51.6 ± 6.59 and 16.75 ± 3.69 μg protein/g at 24 h and 48 h post-injection, respectively (unpublished results). *M. complanata* is a cnidarian that is common in the coral reefs of the Mexican Caribbean and characterized by its capacity to induce local toxic effects in humans that include severe pain, eruptions, and blisters on human skin [26]. In the current study, we found that the extracts from the three scleractinian corals were lethal to crickets, with LD_50_ values similar to those found for *M. complanata* extract. Moreover, the LD_50_ values found for these cnidarians are quite similar to those presented by theraphosid spiders [27].

Cytolysins have been widely reported in several cnidarian venoms; they induce hemolysis in erythrocytes of many different species [20]. Most of these toxins have been isolated from anemones and they act through the formation of pores in cellular membranes; a number of reviews have described their structural and functional characteristics [22, 28–31]. Cytolytic activity on sheep erythrocytes has been reported previously from aqueous extracts from 57 scleractinian corals collected in the Great Barrier Reef. In that study, it was found that the majority of the extracts were potent lysins, causing more than 90% hemolysis [9]. The results obtained in this study showed that rat erythrocytes were more sensitive to *P. strigosa* and *S. siderea* extracts, while the *P. astreoides* extract was more potent in human erythrocytes. Upon comparison with our previous study on the hemolytic properties of *M. complanata* aqueous extract [6], we found that *P. strigosa* extract was significantly more potent than *M. complanata* in both types of erythrocytes. These results suggest important differences between the hemolytic mechanisms of these reef-forming cnidarians.

Cnidarian toxins, including cytolysins and neurotoxins, have been studied with regard to their putative activity in the cardiovascular system [32]. It was found that the sea anemone pore-forming toxin EqT III binds to vascular smooth muscle membranes, causing a slight increase in the tension of the isolated porcine coronary artery rings [33]. The venom from the Portuguese man-of-war (*Physalia physalis*) produces dose-dependent relaxations of norepinephrine-precontracted isolated rabbit arterial rings [34]. The venom of the giant jellyfish *Nemopilema nomurai*, administrated intravenously in rats, induces dose-dependent hypotension and bradycardia (0.1-2.4 mg protein/kg); this venom also produces contraction of isolated rat aortic rings [35]. Tentacle extract from the jellyfish *Cyanea capillata* induces contraction in both endothelium-intact and endothelium-denuded isolated aortic rings [36]. In other studies, it was found that aqueous extracts from the calcareous hydrozoans *M. alcicornis* and *M. complanata* induce vasoconstriction of isolated rat aortic rings [37, 38].

In this study, the three extracts from these scleractinian corals were also found to induce vasoconstriction in isolated rat aortic rings. *P. strigosa* was the most potent, showing an EC_50_ value similar to that previously found for *M. alcicornis* and *M. complanata* [38], both of which are considered toxic to humans [26]. The effects of these scleractinian corals on vascular tone were irregular, since at high concentrations a vasodilator effect appeared to predominate, especially with *P. astreoides* and *S. siderea* extracts, suggesting the presence of vasodilator compounds in the extracts.

Human contact with some cnidarian species can result in moderate to extreme pain. The degree of envenomation depends on the composition of the venom and its method of introduction into the human skin [39]. Severe cases of pain are usually caused by contact with jellyfish, the most dangerous of which is *Chironex fleckeri* [40]. In the present study, evaluation of nociceptive response by the formalin test showed that all three aqueous extracts induced significant nociceptive behavior during the first phase (neurogenic phase). A previous study showed that the transient receptor potential vanilloid channel type 1 (TRPV1), a non-selective cation channel expressed in nociceptive neurons, is a key component in the signal-transduction pathway of pain generated by cnidarian envenomation [41]. However, it is important to mention that peptidic TRPV1 inhibitors have also been isolated from sea anemones, suggesting the presence of analgesic compounds in some cnidarian venoms [42, 43].

In the present work, we have evaluated some of the most common enzymatic activities reported for animal venoms. The presence of PLA_2_ activity has been widely reported in crude extracts and venoms from several cnidarian species [20, 44]. These enzymes have also been detected in venoms of many diverse animals and display a broad spectrum of biological activities. Furthermore, PLA_2_s are considered the major pharmacologically active components of snake venoms [45]. It has been proposed that the presence of PLA_2_ enzymes in cnidarian venoms plays an important role in defense against predators, and in the immobilization and digestion of prey [20, 44]. Moreover, some studies have related PLA_2_ activity to hemolysis [36, 46].

Serine proteases are other enzymes widely reported in animal venoms that not only have been associated with several physiological functions such as platelet aggregation, fibrinolytic activity, spreading activity of other toxins, but also may induce post-translational modifications of other toxins [47–49]. Hyaluronidases are enzymes widely reported in venoms that are not toxic by themselves, but are known as “spreading factors” because they promote the diffusion of toxins through the tissues of the prey [50]. In a predictive study, potential toxin-encoding genes were identified in the scleractinian coral *Acropora digitifera*; the same study revealed putative PLA_2_, serine protease, and hyaluronidase enzymes in that scleractinian coral [51]. Serine proteases have also been detected in the venom of the jellyfish *Cyanea capillata* by transcriptome analysis [52]. The presence of serine protease and PLA_2_ in the crude venom from tentacles of the jellyfish *Olindias sambaquiensis* was experimentally confirmed, showing that the levels of activity of these enzymes were comparable to those observed in venoms of *Bothrops* snakes [53].

In this study, we experimentally confirm PLA_2_ presence and serine protease activities in the aqueous extracts of the scleractinian corals *P. strigosa*, *P. astreoides*, and *S. siderea*. These extracts did not display hyaluronidase activity. The levels of PLA_2_ activity of these scleractinian corals were similar to those previously reported for the hydrozoans *M. complanata* and *M. alcicornis* [38], whereas the serine-protease activity levels were similar to those observed in jellyfish and snakes [53]. These results suggest that PLA_2_ and serine proteases play an important role in the toxicity of the scleractinian corals.

Analysis by electrophoresis revealed that the extracts contain proteinaceous components with a wide range of molecular weights and some differences in their compositions. The profiles of the extracts changed under reducing conditions, suggesting the presence of oligomeric proteins. Based on studies of other cnidarians, enzymes correspond to masses between 25 and 50 kDa [28, 53], whereas cytolysins are found mainly in the region of 20 kDa [28]. Several reports have shown than cnidarian venoms contain an important mixture of peptides with low molecular weights, mostly between 3 and 7 kDa, that act on voltage-gated sodium and potassium channels [29, 54], the presence of these neurotoxins in the cnidarian venoms is important for paralyzing their prey.

In fact, all anemone species tested have been found to contain toxins that are lethal or paralytic to crabs [55]. Other low-molecular-weight peptides found in anemones affect channels implicated in pain, such as TRPV1 and acid sensing ion channels [54]. Moreover, low-molecular-weight cytolytic toxins also have been found in cnidarians; two peptides with molecular masses of 5.1 and 6.1 kDa were isolated from the sea anemone *Radianthus macrodactylus* [56]. Analysis by MALDI-TOF mass spectrometry of the low-molecular-weight fractions obtained from the aqueous extracts revealed the presence of low-molecular-weight peptides. These fractions were clearly toxic since they were lethal to crickets. Moreover, the low-molecular-weight fractions obtained from *P. strigosa* and *P. astreoides* extracts seem to be more complex and contain peptides that exert vasoconstrictor activity on isolated rat aortic rings.

The aqueous extracts of the scleractinian corals analyzed in this study induce toxicological, pharmacological and biochemical effects similar to those observed with cnidarians that are toxic to humans. For example, most of the biological activities reported in this study for these corals are also induced by the aqueous extract of the hydrozoan fire coral *M. complanata. Millepora* species possess two types of nematocyst capable of penetrating human skin, macrobasic mastigophores and stenoteles [57]. In the current study we observed that these scleractinian corals possess mainly holotrich and p-rhabdoid nematocysts; however, a detailed study by electron microscopy is necessary for a proper identification.

Studies on other scleractinian corals have shown that those corals present the four basic morphological patterns of cnidae found in Hexacorallia: spirocysts, holotrich, b- and p-rhabdoids [58]. These observations suggest that the fact that these corals are not considered toxic to humans may be due to a lack of nematocysts capable of penetrating human skin. It is important to mention that the method previously used for the isolation of nematocysts from *Millepora* species did not work with these scleractinian corals. Thus, the nematocysts were directly observed from small samples of soft tissues of the corals. This difficulty to obtain extracts of nematocysts from these corals explains the scarcity of studies on the toxicity of these organisms.

## Conclusions

In summary, the results of this study show that the aqueous extracts of the scleractinian corals *P. strigosa*, *P. astroides*, and *S. siderea* are composed of cytolysins capable of lysing human and rat erythrocytes, toxins that induce nociceptive responses, and enzymes such as serine proteases and PLA_2_ that undoubtedly play an important role in the toxicity of these corals. This study also has suggested that these corals contain low-molecular-weight peptides that are lethal to crickets. Moreover, this study suggests that the scleractinian corals can also produce low-molecular-weight vasoconstrictor peptides. Determining the identity and action mechanism of these peptides is a very important step to pursue.
